# A case series of venous thromboembolic disease in a semi-urban setting in Cameroon

**DOI:** 10.1186/s13104-019-4092-8

**Published:** 2019-01-18

**Authors:** Clovis Nkoke, Denis Teuwafeu, Alice Mapina, Cyrille Nkouonlack

**Affiliations:** 1 Buea Regional Hospital, Buea, Cameroon; 2 Clinical Research Education, Networking and Consultancy, Douala, Cameroon

**Keywords:** Venous thromboembolism, HIV infection, Tuberculosis, Cameroon

## Abstract

**Objective:**

Our goal was to study the clinical characteristics, risk factors and outcome of patients admitted for venous thromboembolism (VTE) in the medical unit of the Buea Regional Hospital, Cameroon between 1st July 2016 and 30th November 2017.

**Results:**

Twenty-two patients were admitted for VTE. There were 12 (54.4%) men. The mean age was 54.9 ± 13.9 years (range: 31–77 years). The main risk factors were immobilization (40.9%), HIV infection (22.7%), tuberculosis (18.2%), obesity (13.6%) and cancer (13.6%). Nineteen (86.4%) patients had deep venous thrombosis (DVT), 3(13.6%) had pulmonary embolism (PE). One patient had a concomitant DVT and PE. All patients received low molecular weight heparin and 76.2% were discharged on oral vitamin K antagonist while 23.8% were discharged on direct oral anticoagulants. The median length of hospital stay was 9.5 days (range: 4–34). Three deaths (13.6%) were recorded. This study describes VTE in a semi-urban setting in Cameroon and shows that immobility, HIV infection and tuberculosis are common risk factors for VTE in this semi-urban setting.

## Introduction

Venous thromboembolism (VTE) includes deep venous thrombosis (DVT) and pulmonary embolism (PE). It is a significant cause of morbidity and mortality [1]. There are many known risk factors for VTE [1]. Infections are among the risk factors for VTE. HIV infection is increasingly being recognized as an independent risk factor for VTE and tuberculosis has also been associated with VTE [2, 3]. These two diseases have the greatest burden in developing countries including Sub-Saharan Africa [4, 5]. These two infections have not been included in risk assessment scores for VTE. The burden of venous thromboembolic disease is growing worldwide with the recognition of new risk factors. There is limited data on venous thromboembolic disease in semi-urban and rural settings in Cameroon. The aim of our study was to report on the characteristics, risk factors and outcome of patients admitted with VTE in the Buea Regional hospital, a semi-urban setting in Cameroon.

## Main text

### Methods

#### Study design and setting

This was a retrospective review carried out in the medical unit of the Buea Regional Hospital, Buea South West region of Cameroon. This is a secondary level Hospital and serves as one of the two main referral centers in the region, with a bed capacity of 111 beds, and a catchment population of about 200,000 inhabitants. The main economic activity in the region is agriculture.

We reviewed the discharge register. All medical records of patients admitted for VTE (DVT/PE) between 1st July 2016 and 30th November 2017 were extracted and reviewed. We extracted data on sex, age, risk factors, treatment length of stay and vital status. Diagnosis of DVT was made using compression ultrasonography and the diagnosis of PE was made using computed tomography. Deep venous thrombosis was diagnosed when there was absent compressibility of veins of the lower extremities or direct visualization of a clot. The diagnosis of PE was made when there was a filling defect in the pulmonary artery or its branches. During the study period, 1440 patients were admitted in the medical unit. The study was approved by the administrative authorities of the Buea Regional Hospital acting as the local ethics committee.

#### Statistical analysis

The data collected was analyzed using SPSS version 22 for windows. Descriptive statistics was performed. The results we reported as counts, percentages, means and standard deviation.

### Results

**Fig. 1:**
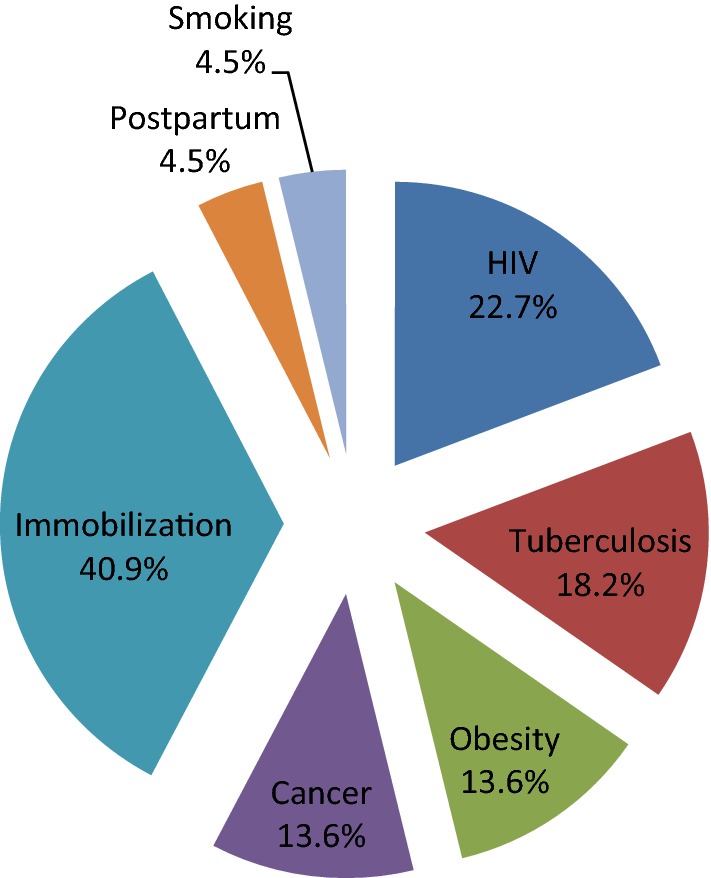
Risk factors of venous thromboembolism

VTE was identified in 22 (1.5%) patients admitted during the study period. There were 12 (54.4%) males. The mean age of the patients was 54.9 ± 13.9 years. Risk factors for VTE identified are shown in Fig. 1. The main risk factor identified was immobilization (9, 40.9%). HIV infection and tuberculosis were identified in 5 (22.7%) and 4 (18.2%) respectively. There were 19 (86.4%) patients who had DVT and 3 (13.6%) patients had PE. Deep venous thrombosis was on the left lower limb in 14 (63.6%) patients. One patient had concomitant DVT and PE. All patients received low molecular weight heparin and 17 (76.2%) were discharged on oral vitamin K antagonist while 5 (23.8%) were discharged on direct oral anticoagulants. The median length of stay was 9.5 days (range: 4–34 days). Three patients with DVT died suddenly, presumably from PE, resulting in a case fatality rate of 13.6%.

### Discussion

We have reported on the characteristics, risk factors and outcome of patients admitted for VTE in Buea, a semi-urban setting in Cameroon. This is the first report on VTE in this region of the country. Our results show that immobilization, HIV infection and tuberculosis were the main risk factors for VTE. Deep venous thrombosis was more common than PE.

Increasing age is one of the main acquired risk factor for VTE. The mean age of patients in our study was 54.9 years. This was similar to that reported by Abah et al. [6] in Bamenda, another semi-urban setting in the North West region of Cameroon. It was however higher than that reported by Kingue et al. [7] in the capital city of Cameroon. The male predominance in our study was similar to that reported by Kingue et al. (55%) and Kamdem et al. (53.8%) in the two major urban cities of Cameroon [7, 8]. In South Africa, Goldstein et al. [9] reported a mean age of 40 years which was lower than that in our study. The younger age in their study is most likely related to the higher rate of HIV infection in this age group as close to half of their patients were HIV positive.

Immobilization was the most common risk factor associated with VTE, followed by HIV infection and tuberculosis in our study. In their study in Bamenda, Abah et al. [6] identified immobilization as the leading risk factor for VTE. Immobilization is a well established risk factor for VTE included in many risk assessment scores for VTE. HIV infection was the second most common risk factor for VTE in our study (22.7%). On the contrary, in South Africa, HIV infection was the most commonly associated risk factor for VTE with 50% of the patients having HIV infection [9]. In an urban city in Cameroon, Kamdem et al. [8] reported a prevalence of HIV infection of 14.1%. HIV infection is increasingly being recognized as a significant risk factor for VTE as suggested by current epidemiological data [2, 10]. Chronic HIV infection is associated with a two–tenfold increase in the risk of VTE. Sub-Saharan Africa has only about 12% of the world’s population, yet accounts for 71% of the global burden of HIV infection [11]. Despite this increased risk of VTE in HIV infection, this risk factor has not been included in major scoring systems for VTE [12–14]. Tuberculosis was another important risk factor for VTE in our study with 18.2% of the patients infected with tuberculosis. This was significantly higher than that reported by Kamdem et al. [8] in the urban city of Douala. This may be due to differences in the epidemiology of tuberculosis between urban and rural settings. The Demographic and Health Survey (DHS) of 2011 in Cameroon estimated the prevalence of HIV to be around 4.3% [15]. Meanwhile the incidence rate of tuberculosis in Cameroon in 2009 was estimated at 182 cases per 100,000 inhabitants [16]. The median length of stay in our study was 9.5 days, which was lower than the 19 days reported by Abah et al. [6] in another semi-urban city in Cameroon. In hospital mortality in our study was 13.6% which was higher than the 10% reported by Kamdem et al. [8] in the urban city of Douala. Abah et al. [6] reported a high in hospital mortality of 21.5% in the city of Bamenda.

### Conclusion

This study describes VTE in a semi-urban setting in Cameroon and shows immobility, HIV infection and tuberculosis are common risk factors associated with VTE in this semi-urban setting.

## Limitations

This study is limited by the small sample size. Also we could not confirm how many cases were investigated for possible DVT/PE as the hospital did not have a diagnostic coding system. However it is the first study to provide insight into the profile of VTE in this part of the country.

## Abbreviations

DVT: deep venous thrombosis
HIV: Human Immunodeficiency Virus
PE: pulmonary embolism
VTE: venous thromboembolism
